# Intravascular Lithotripsy-Assisted Transfemoral Transcatheter Aortic Valve Implantation in Patients with Severe Iliofemoral Calcifications: Expanding Transfemoral Indications

**DOI:** 10.3390/jcm13051480

**Published:** 2024-03-04

**Authors:** Matthias Linder, David Grundmann, Caroline Kellner, Till Demal, Lara Waldschmidt, Oliver Bhadra, Sebastian Ludwig, Lisa Voigtländer, Ina von der Heide, Nicole Nebel, Laura Hannen, Johannes Schirmer, Hermann Reichenspurner, Stefan Blankenberg, Lenard Conradi, Niklas Schofer, Andreas Schäfer, Moritz Seiffert

**Affiliations:** 1 Department of Cardiology, University Heart and Vascular Center Hamburg, Martinistraße 52, 20246 Hamburg, Germany; 2 Department of Cardiology, Regio Kliniken Pinneberg GmbH, Fahltskamp 74, 25421 Pinneberg, Germany; 3 Centre for Population Health Innovation (POINT), University Heart and Vascular Center Hamburg, University Medical Center Hamburg-Eppendorf, 20246 Hamburg, Germany; 4 Department of Cardiovascular Surgery, University Heart and Vascular Center Hamburg, Martinistraße 52, 20246 Hamburg, Germany; 5 German Center for Cardiovascular Research (DZHK), Partner Site Hamburg/Lübeck/Kiel, 24105 Kiel, Germany; 6 Department of Cardiology and Angiology, BG University Hospital Bergmannsheil, Ruhr-University Bochum, Bürkle de la Camp-Platz 1, 44789 Bochum, Germany

**Keywords:** aortic stenosis, TAVI, IVL, intravascular lithotripsy, transfemoral, transaxillary

## Abstract

(1) **Background:** Transfemoral transcatheter aortic valve implantation (TAVI) has become the standard treatment for most patients with severe symptomatic aortic stenosis. Intravascular lithotripsy may facilitate transfemoral TAVI (IVL-TAVI) even in patients with severely calcified iliofemoral disease. We assessed technical aspects and clinical outcomes of this novel approach compared to alternative transaxillary access (TAX-TAVI). (2) **Methods:** IVL-TAVI was performed for severe iliofemoral calcifications precluding standard transfemoral access in 30 patients from 2019 to 2022 at a single academic heart center. IVL was performed as part of the TAVI procedure in all cases. Results were compared to a control group of 44 TAX-TAVI procedures performed for the same indication from 2016 to 2021. The safety outcome was a composite of all-cause death, stroke, access-related bleeding ≥ type 2 within 24 h and major vascular access site complications at 30 days. The efficacy outcome was defined as a technical success according to VARC-3. (3) **Results:** Median age was 78.2 [74.3, 82.6] years, 45.9% were female and mean STS-PROM was 3.6% [2.3, 6.0]. Iliofemoral calcifications were more severe in the IVL-TAVI vs. TAX-TAVI groups (lesion length: 63.0 mm [48.6, 80.3] vs. 48.5 mm [33.1, 68.8]; *p* = 0.043, severe calcification at target lesion: 90.0% vs. 68.2%; *p* = 0.047, and median arc calcification 360.0° [297.5, 360.0] vs. 360.0° [180.0, 360.0]; *p* = 0.033). Technical success was achieved in 93.3% vs. 81.8% (*p* = 0.187) in IVL- and TAX-TAVI and the safety outcome occurred in 10.0% vs. 31.8% in IVL- and TAX-TAVI (*p* = 0.047), respectively. (4) **Conclusions:** IVL-assisted transfemoral TAVI was feasible and safe with favorable outcomes compared to TAX-TAVI. IVL may further expand the number of patients eligible for transfemoral TAVI and may help overcome limitations of an alternative access.

## 1. Introduction

Transfemoral [TF] transcatheter aortic valve implantation [TAVI] has become the treatment of choice for most patients with severe symptomatic aortic stenosis following current guidelines [1]. For patients with severely calcified iliofemoral disease, alternative access routes have been explored. While the transapical approach was favored in the early TAVI years, the transaxillary [TAX] route has become the preferred alternative in most centers in case of unfavorable transfemoral access. TAX may be less invasive than transapical access, but potential drawbacks, compared to the transfemoral route, include higher rates of periprocedural strokes [2]. In addition, noninferiority or even superiority to surgical aortic valve replacement has only been demonstrated for TF-TAVI [3,4,5]. Hence, it appears worthwhile to push TF access for TAVI even in patients with hostile iliofemoral anatomy. Intravascular lithotripsy [IVL], which has been used increasingly in coronary procedures, may be safe and feasible to modify iliofemoral calcifications [6,7,8]. This technology relies on acoustic shock waves to fracture superficial and deep calcium and may be less aggressive compared to high pressure balloon inflation. A reduced risk of barotrauma-induced vessel perforation and/or dissection has been postulated, however clinical data investigating this important topic remain scarce.

Our aim was to evaluate if IVL can facilitate TF access for TAVI in patients with unfavorable femoral routes while avoiding the drawbacks of the TAX approach as an alternative access. Therefore, we assessed technical aspects and clinical outcomes of IVL-facilitated TAVI (IVL-TAVI) and compared them to our previous standard alternative approach, TAX-TAVI.

## 2. Methods

### 2.1. Patient Population and Workup

From 2016 to 2022, a total of 3008 consecutive patients were treated with TAVI for severe aortic valve disease at the University Heart and Vascular Center Hamburg, Germany. In 84 of these patients, coexisting hostile vascular anatomy precluded straightforward TF-TAVI. These patients were either treated with IVL-assisted TF-TAVI (n = 30; 2019–2022) or TAX-TAVI (n = 44 with severe iliofemoral calcification; n = 10 without severe iliofemoral calcification were not included; 2016–2021) (see Supplemental Figure S1). To compare both approaches, we compared outcomes after IVL-assisted TF-TAVI or TAX-TAVI in patients with severe iliofemoral calcifications.

Vascular CT assessment was performed using a dedicated imaging software (3mensio version 10.2, Pi Medical, Amsterdam, The Netherlands). For every lesion, mean reference diameter, target lesion diameter, relative stenosis, lesion length and maximal arc calcification was measured on CT-angiogram. Vascular calcification and tortuosity severity was graded from 0 (none) to 3 (severe) as described before [9].

### 2.2. TAVI Procedures

**Figure 1:**
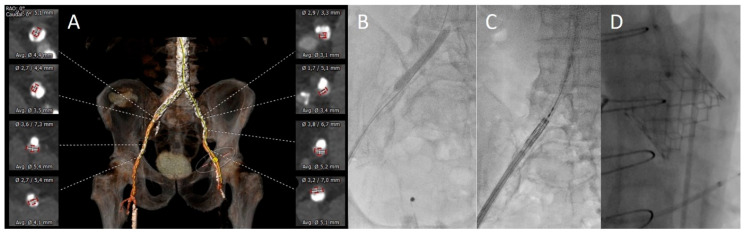
Case example: IVL-assisted TAVI. (**A**): 3dimensional MDCT reconstruction of iliofemoral calcifications and vascular diameters. (**B**): Elective lithotripsy of the right iliofemoral artery (8 × 60 mm IVL balloon with 210 pulses). (**C**): Successful delivery of a 26 mm Sapien Ultra THV through a 14Fr eSheath. (**D**): Implantation of a 26 mm Sapien Ultra with good angiographic result.

**Figure 2:**
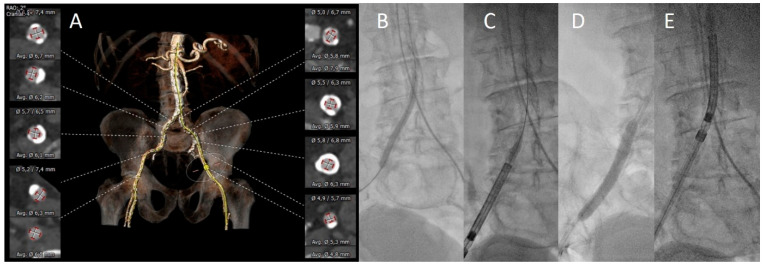
Case example: Bail-out IVL-assisted TAVI. (**A**): Three-dimensional MDCT reconstruction of iliofemoral calcifications and vascular diameters. (**B**): PTA with a 7 mm balloon. (**C**): Unsuccessful delivery of a Evolut R 34 mm. (**D**): Lithotripsy of the right iliofemoral artery (7 × 60 mm, 240 pulses) as bailout strategy. (**E**): Successful delivery of the THV after lithotripsy.

TAX-TAVI was performed in a standard fashion as described before [10,11]. In the IVL group, intravascular lithotripsy of the iliofemoral access route was performed with the Shockwave M5 or M5+ catheter (Shockwave Medical, Santa Clara, CA, USA). IVL was either performed upfront or as a bail-out strategy in case of an unsuccessful delivery of the introducer sheath or the transcatheter heart valve delivery system (see Figure 1 and Figure 2 for case examples). The decision to perform IVL upfront was based on the operators’ MDCT assessment of the stenosis severity, circumferential and longitudinal extension of calcification and tortuosity of the iliofemoral vessels. IVL balloon size was selected to approximate a balloon-to-artery ratio of 1.0 to ensure adequate wall contact for sufficient calcium modification. After performing IVL, the TAVI delivery system was delivered successfully in all cases without any necessity for further postdilatation of the iliac arteries with semi- or non-compliant balloons. After gaining access, TAVI procedures were performed in a standardized manner and under sufficient anticoagulation with unfractionated heparin to achieve an active clotting time >250 s. Following valve implantation, the access route was evaluated with angiography to rule out vascular injuries. Vascular closure was subsequently performed with either suture- or plug-based percutaneous vascular closure devices.

### 2.3. Clinical Endpoints and Data Analysis

All patients’ baseline characteristics, procedural details and clinical outcomes were entered into a dedicated database. Clinical outcomes were adjudicated according to the updated VARC-3 criteria [12].

We defined two primary endpoints:

Efficacy was defined according to the VARC-3 definition of “technical success”. In this combined endpoint, (i) freedom from mortality, (ii) successful access, delivery, and correct positioning of a single THV as well as (iii) freedom from surgery or interventions related to the device or to a major complication at exit from procedure room were required.

If one or more of the following events occurred within 30 days after TAVI, the composite safety endpoint was met: (i) all-cause mortality, (ii) disabling or non-disabling stroke, (iii) major vascular access-site complications and access-related bleeding ≥ type 2 within 24 h after the procedure, as defined in the VARC-3 criteria.

All patients consented to data acquisition as part of the HARbOR clinical cohort. The study was performed in accordance with the 1964 Declaration of Helsinki and its later amendments.

### 2.4. Statistical Analyses

Continuous variables are shown as medians (25th percentile, 75th percentile) and compared using the Kruskal–Wallis test. Binary variables are shown as counts (frequencies) and compared using the Fisher’s exact test. All *p*-values had a significance threshold of <0.05.

## 3. Results

### 3.1. Patient Characteristics

Over a period of 7 years, 2.8% of patients presented with severe aortic stenosis and a hostile vascular anatomy. Their median age was 78.2 [74.3, 82.6] years, 45.9% were female and median STS-PROM was 3.6% without any significant differences in baseline parameters among the IVL- or TAX-TAVI groups. A history of peripheral artery disease was present in 80.0% and 72.7% in the IVL and TAX patients (*p* = 0.581). Previous stroke had occurred in 23.3% and 9.1% (*p* = 0.108), while atrial fibrillation was diagnosed in 46.7% and 25.0% (*p* = 0.079) of patients, respectively (see Table 1).

### 3.2. Vascular Assessment

No significant differences were observed regarding mean reference vessel diameters, minimal target lesion diameters, diameter stenosis or vessel tortuosity. Severe calcification at the target lesion was seen more often in IVL vs. TAX patients (90.0% vs. 68.2%, *p* = 0.047). Correspondingly, lesions had a higher maximal circumferential calcium angle (360.0° [297.5, 360.0] vs. 360.0° [180.0, 360.0]; *p* = 0.033) and were longer (63.0 mm [48.6, 80.3] vs. 48.5 mm [33.1, 68.8]; *p* = 0.043) in the IVL group (see Table 2).

### 3.3. Procedural Aspects

Successful delivery of the transcatheter heart valve was achieved in all patients of both groups. In the IVL group, more procedures were performed in local anesthesia (86.7% vs. 56.8%, *p* = 0.009), while large sheath diameters (≥16 Fr) were more frequent in the TAX group (19.2% vs. 70.5%; *p* < 0.001). Cerebral protection devices were only used in selective cases in TAX patients (6 of 44 TAX cases) and none in IVL-TAVI. Overall, 6.3% were scheduled valve-in-valve procedures and balloon-expandable transcatheter heart valves were implanted in 43.3% vs. 27.3% (*p* = 0.211) of IVL vs. TAX patients.

In the IVL group, 19 cases were planned as IVL-assisted TAVI upfront. In the remaining 11 procedures, IVL was used as a bail-out strategy after failure to advance the introducer sheath or the valve delivery system. In all cases, IVL was performed as a part of the TAVI procedure. The 7 × 60 mm IVL balloon was used in 29 cases and the 8 × 60 mm IVL balloon in one case, respectively. A median of 257 pulses was applied at the iliofemoral arteries.

Vascular closure was mostly plug-based in the IVL group (65.5% vs. 25.6%; *p* = 0.001), while most TAX procedures were closed with a suture-based system (31.0% vs. 74.4%, *p* < 0.001). Surgical cutdown was utilized to gain TF access in one IVL-patient due to massive vascular calcifications at the location of preferred vessel puncture at the common femoral artery. While mean procedure times were similar among both groups, less contrast agent (179.0 mL [135.7, 197.3] vs. 220.0 mL [171.1, 303.3]; *p* = 0.004) and less fluoroscopy (29.5 min [20.9, 36.1] vs. 36.1 min [28.4, 45.0]; *p* = 0.008) were necessary in IVL-assisted TAVI (see Table 3).

### 3.4. Outcome

**Figure 3:**
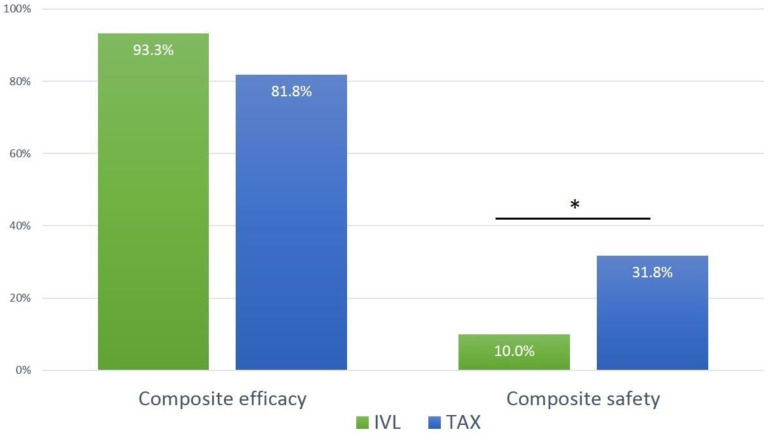
Primary endpoints. Legend: Efficacy was defined as technical success according to the VARC-3 criteria, including freedom from mortality, successful access and positioning of a single THV and freedom from surgery or interventions related to the device or to a major complication. Safety was defined as a composite of all-cause mortality, disabling or non-disabling stroke, major vascular access-site complication and access-related bleeding ≥ type 2 according to VARC-3 at 30 days. * *p* = 0.047.

The composite efficacy endpoint occurred in 93.3% of the IVL group and in 81.8% of the TAX group (*p* = 0.187). The composite safety endpoint occurred less often in the IVL group (10.0% vs. 31.8%; *p* = 0.047) (see Table 4 and Figure 3). Major access site complications were more frequent in the TAX group (3.3% vs. 20.5%; *p* = 0.042), mostly related to bleeding from the axillary access requiring implantation of a covered stent. In the IVL group, one stent implantation was performed at the target lesion in the common iliac artery and one patient required staged vascular surgery after embolization of a plug-based vascular closure system. No major dissections were observed; two minor dissections without flow limitation were treated conservatively. In the TAX group, 11 patients (25.0%) suffered from bleeding complications at the primary vascular access location, requiring a covered stent implantation. In three (6.8%) additional patients, hemostasis was achieved with temporary endovascular balloon inflation at the puncture site; no thrombin injection was performed in this group. In the IVL-TAVI group, no covered stent was required at the primary vascular access location. Four (13.3%) patients developed pseudoaneurysms of whom in one patient underwent thrombin injection. In two patients (6.6%), temporary endovascular balloon inflation was necessary to gain hemostasis. In one other patient, a second plug-based vascular closure device was used subsequent to the failure of the primary closure device. Death at 30 days, stroke and ≥ type 2 bleeding did not differ significantly among both groups with numerically higher rates in TAX patients (see Table 4).

## 4. Discussion

We investigated the technical aspects and clinical outcomes of IVL-TAVI in patients with hostile iliofemoral anatomies and compared them to our preferred alternative access, TAX-TAVI. Our main findings were as follows: IVL-assisted transfemoral TAVI proved (i) feasible and safe in our series of patients with severely calcified access routes and was associated with (ii) favorable outcomes compared to TAX-TAVI.

Transfemoral TAVI has become the standard of care for the treatment of severe aortic stenosis in the majority of patients according to recent treatment guidelines [1]. However, this recommendation relies on the ability to perform the procedure through TF access [3,4,5]. Despite all technical advances regarding introducer sheaths and valve delivery systems, a relevant number of patients present with severe iliofemoral disease impeding straight-forward TF access. While this included historically about 10–15% of patients scheduled for TAVI [13,14], we found this number to be lower, at 2.8%, in our series, which may be more reflective of the current state of treatment. These patients have mostly been treated via alternative access. However, special skillsets and tools required to gain alternative access increased invasiveness, and additional periprocedural complications make it worthwhile to evaluate TF access even in patients with unfavorable anatomies. In addition, severe iliofemoral disease is associated with other comorbidities, rendering these patients at an increased risk for periprocedural complications, irrespective of vascular access-related issues. Moreover, the TF approach can usually be performed with a “minimalist setting” under local anesthesia without any systemic analgesia, safe percutaneous vascular closure, fast recovery, mobilization and early discharge [15,16].

Percutaneous transluminal angioplasty (PTA) has been used in selected cases to modify iliofemoral disease and facilitate TF-TAVI. This approach has been shown to be feasible and safe in most procedures [17]. Whether a relative downsizing of balloon diameter (e.g., a balloon-to-artery ratio <0.9) may be beneficial in reducing vessel injuries remains to be determined. However, distinct vascular complications were noted in few patients that were related to the vascular access in this study [17].

IVL is a novel technique to obtain lumen gain and increase vessel compliance by using sonic pressure waves to disrupt deep and superficial calcium at the same time [18]. Vessel compliance is a critical component of arterial distensibility and a major determinant for the successful passage of the valve delivery system through diseased iliofemoral segments. Whether IVL may be beneficial compared to PTA in modifying this disease, particularly in severely calcified vessels, remains to be determined. However, from a theoretical standpoint, the local application of shockwaves after low-pressure balloon inflation may be less traumatic than high-pressure balloon inflation, yielding less vessel injuries. In a randomized controlled trial comparing IVL and PTA in femoral disease, IVL achieved a greater lumen gain and lower rates of both a need for stent implantation and flow limiting dissections than PTA alone after treating superficial femoral arteries [8]. IVL has also been used to optimize underexpanded iliac or coronary stents. Even though in coronary arteries IVL did not significantly affect the integrity of drug-eluting stents and their polymers in bench testing [19], the peripheral sonic waves are more intense and their effect on freshly implanted stents remains unknown. Hence, bail-out iliac stenting with subsequent IVL for optimization of underexpanded segments remains off-label use.

Given the potential advantages, it appears intuitive to employ the IVL technology to facilitate TF-TAVI in hostile anatomies. However, the evidence for this approach remains scarce. After the first published case report [6], only two larger series have been published to date demonstrating the feasibility and safety of this approach [20,21]. These data were in line with high technical success rates observed in our patient sample, reflected by a procedural success rate of 98.2% and successful transfemoral aortic valve delivery in 100% of cases despite the severity of the disease. Our study adds by comparing these results to those achieved in comparable patients with similar anatomic characteristics by performing TAVI through a percutaneous transaxillary route. With a similar efficacy, we were able to demonstrate a better safety profile following a composite endpoint of clinically relevant vascular events. In addition, IVL procedure duration tended to be shorter (statistical trend) with less contrast and lower radiation doses compared to the TAX approach. Of note, the overall incidence of vascular and bleeding complications remained high in both groups, emphasizing the high procedural risk of patients with calcified iliofemoral disease. Interestingly, the majority of these events occurred at the puncture site. Hence, despite safe passage and modification of the iliofemoral disease in the IVL group without any severe complications, the puncture sites, that are also mostly severely diseased, remain a significant risk factor for vascular events in these patients. This aspect needs to be further investigated, and the refinement of techniques should address this issue. The VARC-3 criteria are very sensitive in detecting minor and major vascular or bleeding complications [12]. While the adverse impact of iliofemoral complications is well-known, others, e.g., a covered stent implantation in the axillary artery, require further research.

Since the decline of transapical access in recent years, TAX has become the main alternative access strategy in most centers. Despite benefits over other alternative access strategies, an elevated periprocedural risk compared to TF has been shown, in particular regarding periprocedural strokes [2,22,23]. Pushing the TF route with IVL assistance may hence be a valid option in patients with hostile iliofemoral anatomies. Whether there is a threshold of iliofemoral disease severity where alternative access may still be beneficial remains to be determined [22]. It also remains unclear which patients particularly benefit from IVL versus standard PTA to modify iliofemoral disease prior to TAVI. Unfortunately, the analysis was not sufficiently powered to evaluate the individual components of the primary endpoint. Nevertheless, the stroke rates for the IVL and TAX patients were in line with previous reports [2,22,23].

### Limitations

The small patient sample, the retrospective and single-center non-randomized design of the study and the unadjusted comparison against an alternative access route from earlier years need to be cautiously taken into consideration when interpreting the data. Hence, these early results remain hypothesis-generating and must be confirmed in larger prospective studies. Nevertheless, despite these limitations, we feel that these acute and early outcomes underline important aspects of novel treatment options that may help improve the care of these high-risk patients undergoing TAVI.

## 5. Conclusions

Transfemoral remains the primary access for TAVI, but hostile anatomies may require alternative access options in selected cases with potential drawbacks. IVL-assisted transfemoral TAVI proved feasible and safe in our series of patients with severely calcified access routes and with favorable outcomes compared to TAX-TAVI. It may offer a promising tool to facilitate transfemoral TAVI even in hostile anatomies, avoiding potential drawbacks of alternative access. Patients with severe iliofemoral disease undergoing TAVI remain a high-risk cohort with specific complication rates, particularly at the puncture site.

## Figures and Tables

**Table 1:** Patient characteristics.

	All (n = 74)	IVL (n = 30)	TAX (n = 44)	*p*-Value
Age, years	78.2 [74.3, 82.6]	80.0 [76.7, 84.2]	77.7 [73.5,82.3]	0.112
Female, n	34 (45.9%)	16 (53.3%)	18 (40.9%)	0.346
Body mass index, kg/m^2^	24.6 [21.7, 27.6]	24.2 [21.6, 27.1]	25.2 [21.8, 28.3]	0.510
STS-PROM, %	3.6 [2.3, 6.0]	3.9 [2.5, 5.5]	3.5 [2.2, 6.0]	0.613
Aortic valve area, cm^2^	0.8 [0.7, 0.9]	0.8 [0.8, 0.9]	0.8 [0.7, 0.9]	1.000
Mean aortic valve gradient, mmHg	30.9 [27.8, 33.9]	30.1 [24.8, 35.5]	31.4 [27.6, 35.2]	0.732
NYHA IV, n	6 (8.3%)	2 (6.9%)	4 (9.3%)	1.000
Arterial hypertension, n	64 (86.5%)	25 (83.3%)	39 (88.6%)	0.514
Severely impaired LVEF (<30%), n	10 (13.5%)	2 (6.7%)	8 (18.2%)	0.187
Glomerular filtration rate, mL/min	52.0 [38.3, 73.6]	52.9 [40.9, 74.1]	37.0 [21.3, 79.3]	0.451
Chronic pulmonary disease, n	25 (33.8%)	9 (30.0%)	16 (36.4%)	0.624
Peripheral artery disease, n	56 (75.7%)	24 (80.0%)	32 (72.7%)	0.585
Atrial fibrillation, n	25 (33.8%)	14 (46.7%)	11 (25.0%)	0.079
History of stroke, n	11 (14.9%)	7 (23.3%)	4 (9.1%)	0.108
Diabetes, n	17 (23.0%)	8 (26.7%)	9 (20.5%)	0.581
Coronary artery disease, n	57 (77%)	23 (76.7%)	34 (77.3%)	1.000

IVL, intravascular lithotripsy; TAX, transaxillary; STS-PROM society of thoracic surgeons predicted risk of mortality score; NYHA, New York Heart Association; LVEF, left ventricular ejection fraction.

**Table 2:** Vascular assessment.

	All (n = 74)	IVL (n = 30)	TAX (n = 44)	*p*-Value
Severe tortuosity of iliofemoral vessels, n	16 (21.6%)	7 (23.3%)	9 (20.5%)	0.781
Severe calcification at target lesion, n	57 (77.0%)	27 (90.0%)	30 (68.2%)	0.047
Reference vessel diameter, mm	7.8 [6.8, 8.9]	7.8 [6.9, 8.6]	7.8 [6.7, 9.4]	0.969
Target lesion: common or external iliac artery, n	69 (93.2%)	29 (96.7%)	40 (90.1%)	0.642
Target lesion diameter, mm	4.3 [3.6, 4.8]	4.4 [3.5, 4.8]	4.2 [3.6, 4.7]	0.567
Diameter stenosis, %	56.2 [43.6, 62.9]	58.2 [46.6, 61.4]	54.5 [42.9, 63.2]	0.560
Target lesion length, mm	54.5 [39.8, 73.1]	63.0 [48.6, 80.3]	48.5 [33.1, 68.8]	0.043
Maximal arc calcification, °	360.0 [262.5, 360.0]	360.0 [297.5, 360.0]	360.0 [180.0, 360.0]	0.033
Circular calcification (360°), n	42 (56.7%)	19 (63.3%)	23 (52.3%)	0.474
Horseshoe-like calcification (270°), n	14 (18.9%)	11 (36.7%)	3 (6.8%)	0.002

IVL, intravascular lithotripsy; TAX, transaxillary.

**Table 3:** Procedural aspects.

	All (n = 74)	IVL (n = 30)	TAX (n = 44)	*p*-Value
Local anesthesia, n	51 (68.9%)	26 (86.7%)	25 (56.8%)	0.010
Sheath size ≥16 Fr, n	34 (50.0%)	5 (19.2%)	29 (69.0%)	<0.001
Cerebral protection, n	6 (8.1%)	0	6 (13.6%)	0.075
Balloon-expandable THV, n	25 (33.8%)	13 (43.3%)	12 (27.2%)	0.211
Planned valve-in-valve, n	4 (6.3%)	1 (5.3%)	3 (6.8%)	1.000
Successful delivery of THV, n	74 (100%)	30 (100%)	44 (100%)	1.000
IVL balloon size 7 × 60 mm/8 × 60 mm, n		29 (96.7%)/1 (3.3%)	-	
Number of pulses, n		257.3 [232.0, 300.0]	-	
Elective/bail-out IVL, n		19 (63.3%)/11 (36.7%)	-	
Vascular closure				<0.001
Suture-based closure system	41 (56.9%)	9 (31%)	32 (74.4%)	<0.001
Plug-based closure system	30 (41.7%)	19 (65.5%)	11 (25.6%)	0.001
Planned surgical vascular access	1 (1.4%)	1 (3.4%)	0	0.403
Procedure time, min	105 [85.0, 130.0]	93.0 [80.0, 124.1]	110.0 [92.1, 130.0]	0.090
Fluoroscopy time, min	34.0 [24.4, 39.2]	29.5 [20.9, 36.1]	36.1 [28.4, 45.0]	0.008
Contrast agent, mL	192.5 [157.7, 260.0]	179.0 [135.7, 197.3]	220.0 [171.7, 303.3]	0.004

IVL, intravascular lithotripsy; TAX, transaxillary; Fr, French; THV, transcatheter heart valve.

**Table 4:** Clinical outcomes.

	All (n = 74)	IVL (n = 30)	TAX (n = 44)	*p*-Value
Composite efficacy endpoint, n (at exit from procedure room)	64 (86.5%)	28 (93.3%)	36 (81.8%)	0.187
Freedom from mortality, n	74 (100%)	30 (100%)	44 (100%)	1.000
Successful access, delivery of the device, and retrieval of the delivery system, n	74 (100%)	30 (100%)	44 (100%)	1.000
Correct positioning of a single THV, n	74 (100%)	30 (100%)	44 (100%)	1.000
Freedom from surgery or intervention related to the device or to a major complication, n	64 (86.5%)	28 (93.3%)	36 (81.8%)	0.187
Composite safety endpoint, n (at 30 days)	17 (23.0%)	3 (10.0%)	14 (31.8%)	**0.047**
All-cause mortality, n	5 (6.8%)	1 (3.3%)	4 (9.1%)	0.642
Disabling or non-disabling stroke, n	5 (6.8%)	1 (3.3%)	4 (9.1%)	0.642
Major vascular access-site complication, n	10 (13.5%)	1 (3.3%)	9 (20.5%)	**0.042**
Access-related bleeding ≥ type 2 <24 h, n	8(10.8%)	1 (3.3%)	7 (15.9%)	0.132
Minor vascular access-site complication, n	20 (27.0%)	8 (26.7%)	12 (27.3%)	1.000
Unplanned treatment due to vascular complication at primary vascular access location				
Covered stent implantation	11 (14.9%)	0 (0%)	11 (25.0%)	**0.002**
Endovascular balloon inflatation	5 (6.8%)	2 (6.6%)	3 (6.8%)	1.000
Thrombin injection	1 (1.4%)	1 (3.3%)	0	0.405
Vascular surgery	4 (5.4%)	1 (3.3%)	3 (6.8%)	0.642
IVL-specific outcome				
Perforation, n		0	-	
Major dissection, n		0	-	
Minor dissection (conservative treatment), n		2 (6.7%)	-	
Stent implantation at IVL location		1 (3.3%)	-	
Myocardial infarction	0	0	0	
Acute kidney injury (stage 3)	3 (4.7%)	1 (5.0%)	2 (4.5%)	1.000

IVL, intravascular lithotripsy; TAX, transaxillary. Bold stands for a main category (e.g. “Composite effifacy endpoint”) and includes the subsequent components (“Freedom from mortality”, “Succesful access” etc.).

## Data Availability

The data presented in this study are available on request from the corresponding author.

## Supplementary Materials

The following supporting information can be downloaded at: https://www.mdpi.com/article/10.3390/jcm13051480/s1, Figure S1: Study flow chart.

## Abbreviations

CT	Computed tomography
IVL	Intravascular lithotripsy
PTA	Percutaneous transluminal angioplasty
STS-PROM	Society of thoracic surgeons—predicted risk of operative mortality
TAVI	Transcatheter aortic valve implantation
TAX	Transaxillary
TF	Transfemoral
