# Supplementary figures and images for: Intravascular Lithotripsy-Assisted Transfemoral Transcatheter Aortic Valve Implantation in Patients with Severe Iliofemoral Calcifications: Expanding Transfemoral Indications
Source: J Clin Med. 2024 Mar 4;13(5):1480. doi: 10.3390/jcm13051480 (PMC10932192; doi:10.3390/jcm13051480)

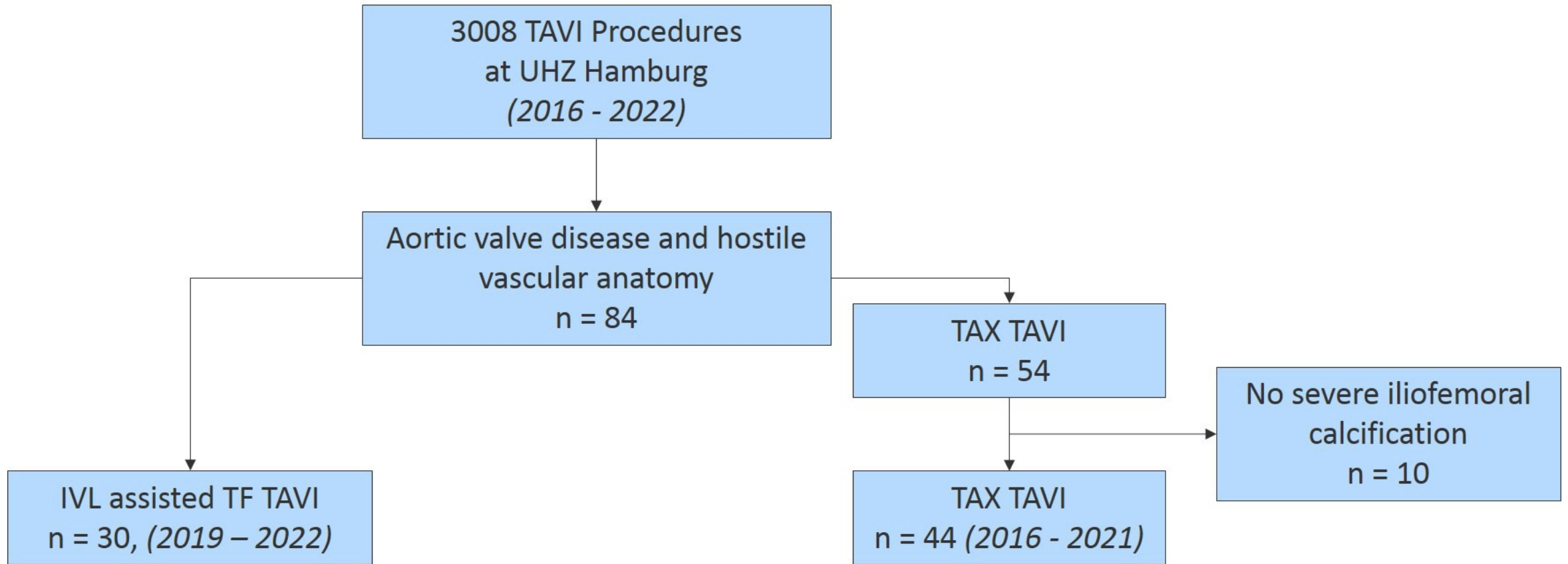

**Supplemental Figure S1:** Study flow chart

Supplement: Supplementary file 1 [file jcm-13-01480-s001.zip › jcm-2884440-supplementary.pdf]
